# Prevalence and Clinical Predictors of Insulin Resistance in Reproductive-Aged Thai Women with Polycystic Ovary Syndrome

**DOI:** 10.1155/2012/529184

**Published:** 2012-01-12

**Authors:** Thanyarat Wongwananuruk, Manee Rattanachaiyanont, Suchada Indhavivadhana, Pichai Leerasiri, Kitirat Techatraisak, Prasong Tanmahasamut, Surasak Angsuwathana, Chongdee Dangrat

**Affiliations:** Gynecologic Endocrinology Unit, Department of Obstetrics and Gynecology, Faculty of Medicine Siriraj Hospital, Mahidol University, Bangkok 10700, Thailand

## Abstract

*Objectives*. To determine the prevalence of insulin resistance (IR) and its predictors in reproductive-aged Thai women with polycystic ovary syndrome (PCOS). *Methods*. A cross-sectional study was conducted from May 2007 to January 2009. Participants were 250 Thai women with PCOS. Information regarding medical history and physical examination and results of 75 gram OGTT were recorded. *Results*. The overall prevalence of IR was 20.0%, comprising the prevalence of impaired fasting glucose, impaired glucose tolerance, and diabetic mellitus of 3.2%, 13.6%, and 5.6%, respectively. Multiple logistic regression analysis showed that the independent predictors for IR were age of ≥30 years old, waist circumference (WC) of ≥80 cm, presence of acanthosis nigricans (AN), and dyslipidemia with odds ratios (95% confidence interval) of 2.14 (1.01–4.52), 3.53 (1.28–9.75), 2.63 (1.17–5.88), and 3.07 (1.16–8.11), respectively. *Conclusion*. The overall prevalence of IR in reproductive-aged Thai women with PCOS is 20.0%. Age ≥30 years old, WC ≥80 cm, the presence of AN, and dyslipidemia are the significant clinical predictors.

## 1. Introduction

Polycystic ovary syndrome (PCOS) is one of the most common endocrine disorders, affecting around 4–7% of reproductive-aged women [1]. The etiology of PCOS remains to be elucidated, although insulin resistance (IR) and hyperinsulinemia seem to be the important mechanism for PCOS and its metabolic derangements [2, 3], diabetes mellitus (DM) in particular [4, 5]. It has been reported that PCOS women are at high risk of IR as shown by various markers, for example, impaired glucose tolerance of 25–31%, and type 2 DM of 7.5–10% [6, 7].

Patients with IR, especially those with DM, have a high risk for developing cardiovascular disease in the future [8, 9]. Although PCOS women have a high risk of IR, the Rotterdam ESHRE/ASRM-sponsored PCOS Consensus Workshop Group does not recommend any test of IR to be routinely used in clinical practice. However, it is suggested that in large-scale clinical or epidemiological studies, simple methods such as fasting plasma glucose (FPG) and oral glucose tolerance test (OGTT) may provide effective estimates for IR [10]. 

In our previous report [11], we found that the PCOS women at our clinic were relatively young and thin as compared with those in other reports [6, 7, 12]. This specific group of PCOS women might have lower risk of IR. The objectives of the present study were to investigate the prevalence and clinical predictors of IR in PCOS Thai women.

## 2. Materials and Methods

The present cross-sectional study was conducted at the Gynecologic Endocrinology Unit, Faculty of Medicine Siriraj Hospital, Mahidol University. The study protocol was approved by the Siriraj Institutional Review Board. 

### 2.1. Participants

Participants were all PCOS Thai women who registered to the Siriraj PCOS project from May 2007 to January 2009 [11]. The diagnosis of PCOS was made according to the revised Rotterdam 2003 criteria [10]. Additional exclusion criteria were women who had previous surgery of one or both ovaries, used hormonal treatment, took the medication for dyslipidemia within 3 months, or took steroid within 6 months before registering to the project. 

After a written informed consent was obtained, a structured medical record form was used to collect the following data: clinical presentation, medical history, age, body weight, height, waist circumference (WC), blood pressure, and skin manifestations. The detailed method of clinical examination was published in our previous report [11]. 

Glucose tolerance was evaluated using 75 g OGTT. Venous blood samples were drawn twice from antecubital vein. The first sample was drawn at 8–10 AM after overnight fasting for at least 12 hours, and the second samples was drawn two hours after 75 g oral glucose loading. The first blood sample was examined for baseline metabolic profiles (fasting blood glucose (FBG), cholesterol, triglyceride, high density lipoprotein cholesterol (HDL-C), low density lipoprotein cholesterol (LDL-C)), and hormonal profiles (total testosterone, prolactin, thyroid stimulating hormone (TSH), and cortisol). The second blood sample was examined for blood glucose and insulin levels.

### 2.2. Definitions

Obesity was defined as body mass index (BMI) ≥25 kg/m^2^ according to the WHO cutoff points for Asian populations [13]. Central obesity was defined as WC ≥80 cm according to the International Diabetes Federation (IDF) 2005 [14]. 

Acanthosis nigricans was a cutaneous condition characterized by a velvety darkening of the skin in the posterior region of neck, axillae, antecubital fossa, groin, and other areas [15]. 

Hypertension was defined as systolic blood pressure of ≥140 mmHg or diastolic blood pressure of ≥90 mmHg , according to the World Health Organization/International Society of Hypertension Writing Group (WHO/ISH) 2003 [16]. 

Dyslipidemia was defined as cholesterol ≥200 mg/dL, LDL-C ≥160 mg/dL, HDL-C <50 mg/dL, or triglyceride ≥150 mg/dL, according to National Cholesterol Education Program Adult Treatment Panel (NCEP ATP) III [17]. 

Clinical hyperandrogenism was considered if the participants had at least one of the followings: hirsutism (a modified Ferriman-Gallwey score ≥8), acne, androgenic alopecia, or virilization. Hyperandrogenemia was defined as total testosterone >0.8 ng/dL, free testosterone >0.006 ng/dL, or dehydroepiandrosterone sulphate (DHEAS) >350 mcg/dL [18]. 

Abnormal plasma OGTT was classified according to the American Diabetes Association (ADA) 2003 criteria [19] as follows: (i) impaired fasting glucose (IFG), that is, fasting blood glucose (FBG) ≥100 and <126 mg/dL, (ii) impaired glucose tolerance test (IGT), that is, 2-hr glucose ≥140 and <200 mg/dL, and (iii) type 2 DM, that is, FBG ≥126 mg/dL or 2-hr glucose ≥200 mg/dL. In the present study, IR was defined as the presence of IFG, IGT, or type 2 DM.

### 2.3. Laboratory Assays

All laboratory assays were performed at the laboratory unit of the Department of Clinical Pathology, Faculty of Medicine Siriraj Hospital, Mahidol University, the central laboratory certified by IS0 15189. All assays were done using automatic analyzers, Modular P800, Roche (for total cholesterol, HDL-C, LDL-C, triglycerides, glucose, and albumin) and Modular E170, Roche (for insulin, TSH, prolactin, cortisol, total testosterone, and DHEAS). Serum-free testosterone was calculated from serum total testosterone, sex hormone-binding globulin (SHBG) and albumin using the online computer program of International Society for the Study of the Aging Male (ISSAM) [20]. All techniques had intra- and interassay coefficients of variation (CV) less than 5%.

### 2.4. Statistical Analysis

Sample size was calculated using a formula for descriptive data. When an estimated prevalence of IR = 20% (from our pilot study), precision error of estimation (25% of *P*) = 0.05, and *α* = 0.05, a sample size of at least 246 was needed.

Statistical analysis was performed using SPSS for Windows, version 11.5. (SPSS Inc.). The normality of distribution of all continuous variables was checked using Kolmogorov-Smirnov test. Data were presented in mean and standard deviation (SD), number (*n*) and percent (%), or odds ratio (OR) and 95% confidence interval (CI), as appropriate. Univariate analysis using Student *t*-test, Mann-Whitney *U* test (for continuous data), or *Chi*-square test or Fisher's exact test (for categorical data) was applied to survey potential predictors of IR. Multiple logistic regression was used to identify the significant predictors. All tests were two-sided, and statistical significance was considered to exist when a *P* value was <0.05.

## 3. Results

Table 1 demonstrates clinical and laboratory characteristics of 250 reproductive-aged Thai women with PCOS. The mean ± SD of age, body mass index (BMI), and waist circumference (WC) were 25.4 ± 5.8 years, 26.2 ± 7.6 kg/m^2^, and 82.3 ± 16.3 cm, respectively. Almost all of the participants (98.4%) presented with oligomenorrhea. Of all participants, 97.2% had ultrasonographic polycystic ovary and 49.2% had hyperandrogenism and/or hyperandrogenemia.

Table 2 shows the prevalence of IR in 250 PCOS Thai women. The overall prevalence was 20.0% which included 3.2% of IFG, 13.6% of IGT, and 5.6% of type 2 DM (3.6% of FBS ≥126 mg/dL, 5.2% of 2-hr glucose ≥200 mg/dL, and 3.2% of combined abnormalities).

Table 3 shows clinical predictors for IR in 250 PCOS Thai women. Comparing with the patients without IR, the ones with IR were older, and had a higher BMI, larger WC, and higher prevalence of hypertension, acanthosis nigricans, and dyslipidemia. 

Table 4 shows the independent predictors for IR in 250 PCOS Thai women. The results from multiple logistic regression analysis by forward stepwise technique showed that the independent predictors for IR included age of ≥30 years old, WC of ≥80 cm, presence of AN, and dyslipidemia, with odds ratios of 2.14 (95% CI 1.01–4.52), 3.53 (95% CI 1.28–9.75), 2.63 (95% CI 1.17–5.88), and 3.07 (95% CI 1.16–8.11), respectively.

## 4. Discussion

Insulin resistance is a metabolic disorder caused by the impairment of insulin function in inducing glucose uptake and utilization. In clinical setting, various techniques are used for the diagnosis of IR [21]. These techniques, including FPG, OGTT, glucose/insulin ratio, homeostasis model assessment for insulin resistance (HOMA-IR), and quantitative insulin sensitivity check index (QUICKI), are shown to correlate well with the gold standard, that is, euglycemic-hyperinsulinemic clamp [22, 23]. To date, it is still inconclusive to judge the superiority of one technique over the others [24, 25]. In the present study, we used FPG and OGTT as suggested by the Rotterdam ESHRE/ASRM-sponsored PCOS Consensus Workshop Group [10]. The overall prevalence of IR in our population was 20.0%. Interestingly 88.2% (30/34) of the patients with IGT and 85.7% (12/14) of those with type 2 DM did not have IFG. Our finding was in line with the previous information that FPG alone is not a sensitive indicator for IR in women with PCOS [6, 7, 26]. 

The prevalence of IR in our PCOS women was lower than that of many reports [6, 7, 27] which revealed the prevalence up to 75%. The difference in techniques used to define IR was responsible for such large discrepancy. However, even using similar definition, the prevalence of IR in our study was still lower than that in other studies [6, 28, 29]. This was owing to the fact of that our study population was relatively young and thin. Previous studies showed that the prevalence of IR increased with age and BMI [6, 7]. 

In the present relatively young and thin PCOS women, many factors associating with IR were similar to those with metabolic syndrome [17, 30] as these two conditions are closely related [31, 32]. These factors included age, central obesity, presence of acanthosis nigricans, and dyslipidemia. Hypertension and high BMI were shown to be associated with IR in univariate analysis but regression analysis indicated that these two variables were not independent factors. Moreover, the association between IR and central obesity but not BMI suggested the effect of body fat distribution on insulin metabolism. Previous studies showed that android fat distribution and upper-half type body fat distribution were linked to insulin resistance in PCOS [33–35]. 

Hyperandrogenemia, a diagnostic criterion for PCOS, was shown to be significantly associated with IR in our univariate analysis. Unexpectedly, the association was not significance in the regression analysis. The discrepancy result between these two analyses indicated that hyperandrogenemia had less impact on IR than other factors did. Previous study demonstrated positive correlations between insulin and androgen levels [36]. Such correlation existed in both obese and nonobese PCOS women [37]. The association between hyperandrogenemia and IR was also demonstrated in previous reports [38, 39]. The pathophysiology of PCOS comprising a model of complex interaction between hyperandrogenemia and IR is well accepted [40]. However, the results of some studies suggested that these two conditions might be two separate entities [38, 41]. The absence of significant association between hyperandrogenemia and IR in the present study might confirm the concept of two separate entities. However, we could not disregard the important of ethnicity on this issue. To date the reference values of androgens in Asian population are not available. The reference values used in the present study derived from the US population, the values of which might not be applicable for our population. 

The results of the present study had to be interpreted with caution. Due to the limitation of a cross-sectional study, the prevalence of IR found at the initial visit would change with time. Therefore, a long-term prospective study is needed to reveal the actual prevalence in our population, and the effect of metabolic derangement on future health status. Furthermore, the majority of our participants were urbanized women; therefore, they might not represent the whole picture of PCOS Thai women.

## 5. Conclusion

The overall prevalence of abnormal OGTT in reproductive-aged Thai women with PCOS is 20.0%. Clinical predictors are age of ≥30 years, central obesity, presence of acanthosis nigricans, and dyslipidemia, but not hyperandrogenemia. It is probably beneficial to screen for IR in these specific groups of PCOS women in order to provide optimum management for each patient.

## Figures and Tables

**Table 1:** Characteristics of 250 Thai women with polycystic ovary syndrome.

Characteristics	Mean ± SD or *n* (%, 95% CI)
Age (yr)	25.4 ± 5.8
Body mass index (kg/m^2^)	26.2 ± 7.6
Waist circumference (cm)	82.3 ± 16.3
Systolic blood pressure (mmHg)	112.5 ± 12.5
Diastolic blood pressure (mmHg)	70.3 ± 9.1
Presence of acanthosis nigricans	68 (27.2, 21.7–32.7)
Clinical criteria	
Oligomenorrhea and/or amenorrhea	246 (98.4, 96.8–100)
Hyperandrogenism and/or hyperandrogenemia	123 (49.2, 43.0–55.4)
Ultrasonographic polycystic ovaries	242 (97.2, 95.1–99.2)
Carbohydrate metabolism	
Fasting plasma glucose (mg/dL)	85.4 ± 22.9
Fasting plasma insulin (mu/mL)	15.6 ± 34.2
2-hour plasma glucose (mg/dL)	116.4 ± 53.8
2-hour plasma insulin (mu/mL)	106.6 ± 89.0
Lipid profiles	
Cholesterol (mg/dL)	189.2 ± 37.6
Triglyceride (mg/dL)	103.2 ± 66.2
High-density lipoprotein cholesterol (mg/dL)	55.4 ± 14.6
Low-density lipoprotein cholesterol (mg/dL)	112.0 ± 32.5
Androgen profiles	
Total testosterone (ng/mL)	0.734 ± 0.387
Free testosterone (ng/mL)	0.014 ± 0.009
Dehydroepiandrosterone sulphate (microgram/dL)	256.8 ± 107.2

**Table 2:** Prevalence of insulin resistance in 250 Thai women with polycystic ovary syndrome.

Insulin resistance^†^	Prevalence	
	*n*	% (95% CI)
Overall	50	20.0 (15.04–24.96)
Impaired fasting glucose (IFG)^ ‡^	8	3.2 (1.02–5.38)
Impaired glucose tolerance (IGT)	34	13.6 (9.35–17.85)
Diabetes mellitus (DM)	14	5.6 (2.75–8.45)
Fasting plasma glucose ≥ 126 mg/dL	9	3.6 (1.29–5.91)
2 hr glucose ≥ 200 mg/dL	13	5.2 (2.45–7.95)
Combined abnormalities	8	3.2 (1.02–5.38)

^†^Insulin resistance: impaired fasting glucose (fasting plasma glucose ≥100 and <126 mg/dL), impaired glucose tolerance test (2-hr glucose ≥140 and <200 mg/dL), or the presence of diabetes mellitus.

^‡^4 women had combined IFG and IGT and 2 women had combined IFG and 2 hr glucose ≥200 mg/dL.

CI: confidence interval.

**Table 3:** Predictors for insulin resistance in 250 Thai women with polycystic ovary syndrome.

Variables	*N*	Presence of insulin resistance	
		*n* (%)	OR (95% CI)
Age, yrs			
<30	193	30 (15.5)	1
≥30	57	20 (35.1)	2.94 (1.50–5.73)
Body mass index, kg/m^2^			
<25	135	10 (7.4)	1
≥25	115	40 (34.8)	6.67 (3.15–14.11)
Waist circumference, cm			
<80	128	7 (5.5)	1
≥80	122	43 (35.2)	9.41 (4.03–21.96)
Acanthosis nigricans			
Absence	182	20 (11.0)	1
Presence	68	30 (44.1)	6.40 (3.28–12.46)
Hypertension^†^			
Absence	234	42 (17.9)	1
Presence	16	8 (50)	4.57 (1.62–12.87)
Dyslipidemia^‡^			
Absence	92	6 (6.5)	1
Presence	158	44 (27.8)	5.53 (2.25–13.58)
Hyperandrogenemia^§^			
Absence	45	4 (8.9)	1
Presence	205	46 (22.4)	2.97 (1.00–8.71)

^†^Hypertension: systolic blood pressure ≥140 mmHg or diastolic blood pressure ≥90 mmHg.

^‡^Dyslipidemia: cholesterol ≥200 mg/dL, high-density lipoprotein cholesterol <50 mg/dL, low-density lipoprotein cholesterol ≥160 mg/dL, or triglyceride ≥150 mg/dL.

^§^Hyperandrogenemia: total testosterone >0.8 ng/dL, free testosterone >0.006 ng/mL, or dehydroepiandrosterone sulphate >350 microgram/dL.

CI: confidence interval; OR: odds ratio.

**Table 4:** Independence predictors for insulin resistance in 250 Thai women with polycystic ovary syndrome.

Variables	OR (95% CI)*
Age ≥ 30 yrs	2.14 (1.01–4.52)
Waist circumference ≥ 80 cm	3.53 (1.28–9.75)
Presence of acanthosis nigricans	2.63 (1.17–5.88)
Dyslipidemia	3.07 (1.16–8.11)

*Data were analyzed using multiple logistic regression analysis with forward stepwise technique. All factors in Table 3 were included in the analysis.

CI: confidence interval; OR: odds ratio.
